# Diagnostic Dilemma: Metastatic Breast Adenocarcinoma Presenting With a Cholestatic Liver Injury Without Radiological Findings of Liver Metastases

**DOI:** 10.7759/cureus.81368

**Published:** 2025-03-28

**Authors:** Obada Daaboul, Genan Arman, Mulham Alom, Aman Swied

**Affiliations:** 1 Internal Medicine, Southern Illinois University School of Medicine, Springfield, USA

**Keywords:** cholestatic liver injury, hyperbilirubinemia, liver biopsy, liver metastases, metastatic breast adenocarcinoma

## Abstract

Cholestasis refers to a reduction in bile flow caused by either impaired secretion or obstruction. Cancers may manifest with cholestasis due to metastasis (either obstruction or infiltration), paraneoplastic syndromes, or as a side effect of treatment (chemotherapy, immunotherapy, or radiation). Various imaging techniques, including ultrasound (US), computed tomography (CT), magnetic resonance imaging (MRI), and positron emission tomography (PET) scans, are typically used to assess the obstruction, its location, and the underlying cause. We are presenting a complex and challenging case of metastatic breast cancer in the liver that presented as cholestatic liver injury without corresponding radiological findings. We aim to highlight the importance of considering liver metastasis as a differential diagnosis, even in the absence of supporting radiological evidence.

## Introduction

Cholestasis arises from the disruption of bile formation and/or bile flow, and it can be categorized into intrahepatic and extrahepatic types [1]. Etiologies of cholestasis vary in the defect's anatomical location and presentation acuity. Acute cholestasis can be caused by various factors, including biliary obstruction, cholangitis, drug-induced liver injury (DILI), sepsis, TPN-associated cholestasis, and intrahepatic cholestasis of pregnancy [2]. The American Gastroenterological Association recommends using the R ratio, calculated as R = (patient's alanine aminotransferase (ALT) / upper limit of normal ALT) / (patient's alkaline phosphatase (ALP) / upper limit of normal ALP), to help differentiate between hepatocellular, cholestatic, or mixed liver injury. An R ratio <2 suggests a cholestatic pattern, which can help differentiate between intrahepatic and extrahepatic causes [3]. Cholestatic jaundice can be a presentation of cancers for various reasons. This may occur due to metastasis through recognized mechanisms, such as bile duct obstruction or extensive liver infiltration. Additionally, it can result from treatment modalities, including chemotherapy (such as palbociclib or combinations of fulvestrant and palbociclib), radiotherapy, and immunotherapy [4-7]. There are also rare paraneoplastic syndromes associated with multiple malignancies (such as renal cell cancer, bronchogenic carcinoma, bladder cancer, pancreatic cancer, metastatic prostate adenocarcinoma, soft tissue sarcoma, and malignant lymphoproliferative diseases) that can present with cholestatic jaundice as well [8]. Imaging modalities such as ultrasound (US), computed tomography (CT), magnetic resonance imaging (MRI), and fluorodeoxyglucose (FDG) positron emission tomography (PET) scans are used to assess the obstruction, location, and etiology based on the clinical context. We are reporting a challenging case of metastatic breast cancer in the liver presenting as cholestatic liver injury without correlating radiological findings. This case was previously presented as an abstract at the 2024 American College of Gastroenterology (ACG) Annual Scientific Meeting on October 28, 2024.

## Case presentation

**Figure 1:**
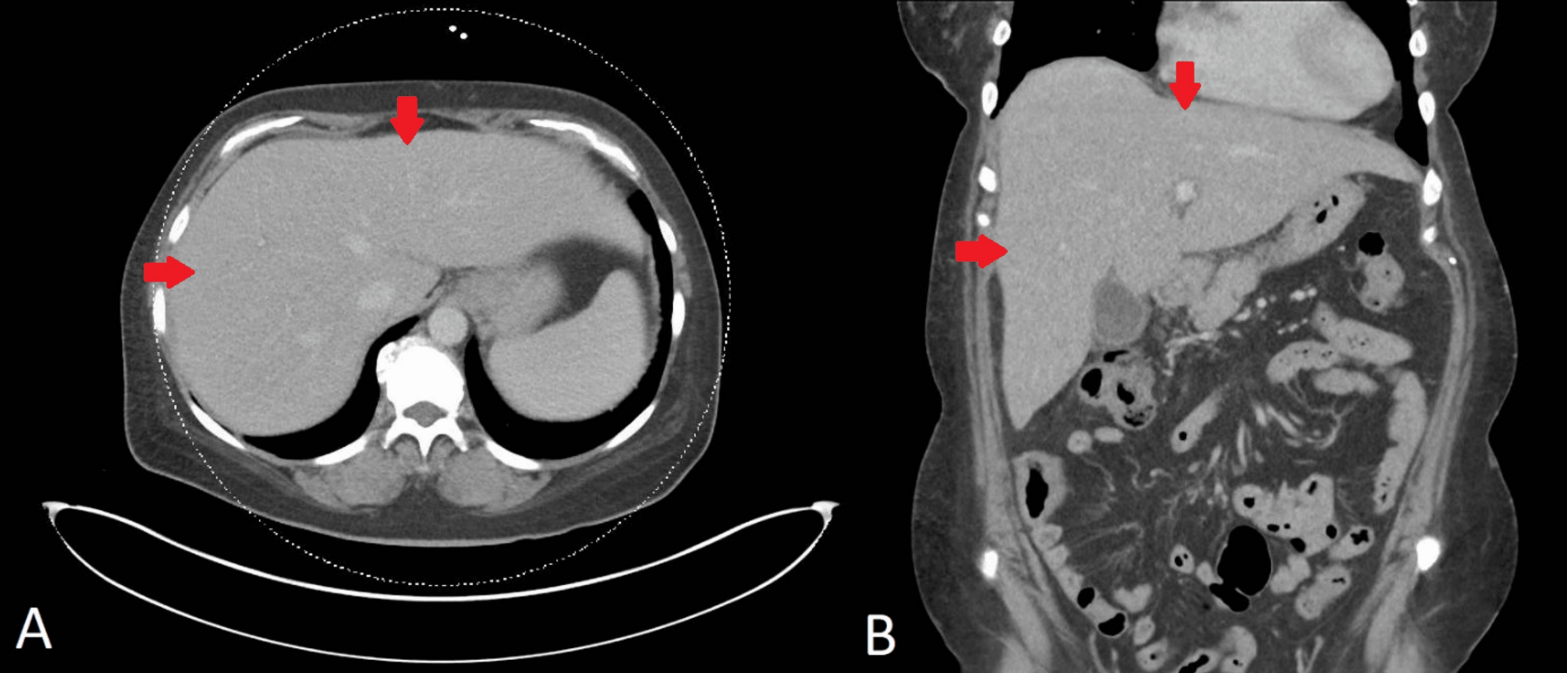
Computed tomography (CT) scan of the abdomen with contrast CT scan of the abdomen with contrast: axial view (A) and coronal view (B) showing homogeneous attenuation and uniform enhancement without focal lesions or any evidence of malignancy

**Figure 2:**
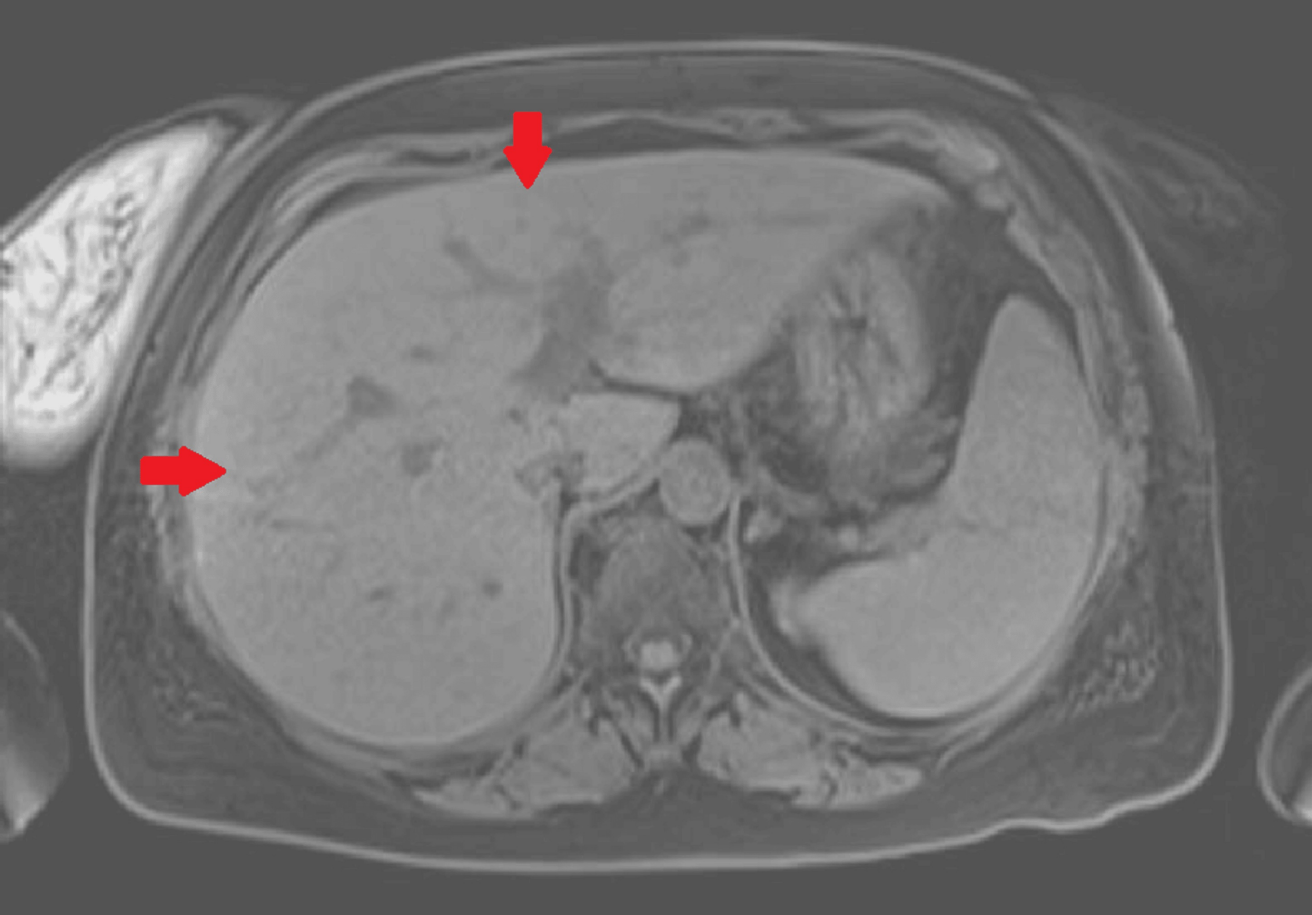
Magnetic resonance imaging (MRI) of the liver Liver MRI demonstrating homogeneous signal intensity and uniform enhancement without any focal lesions or abnormal signal intensities indicative of malignancy

**Figure 3:**
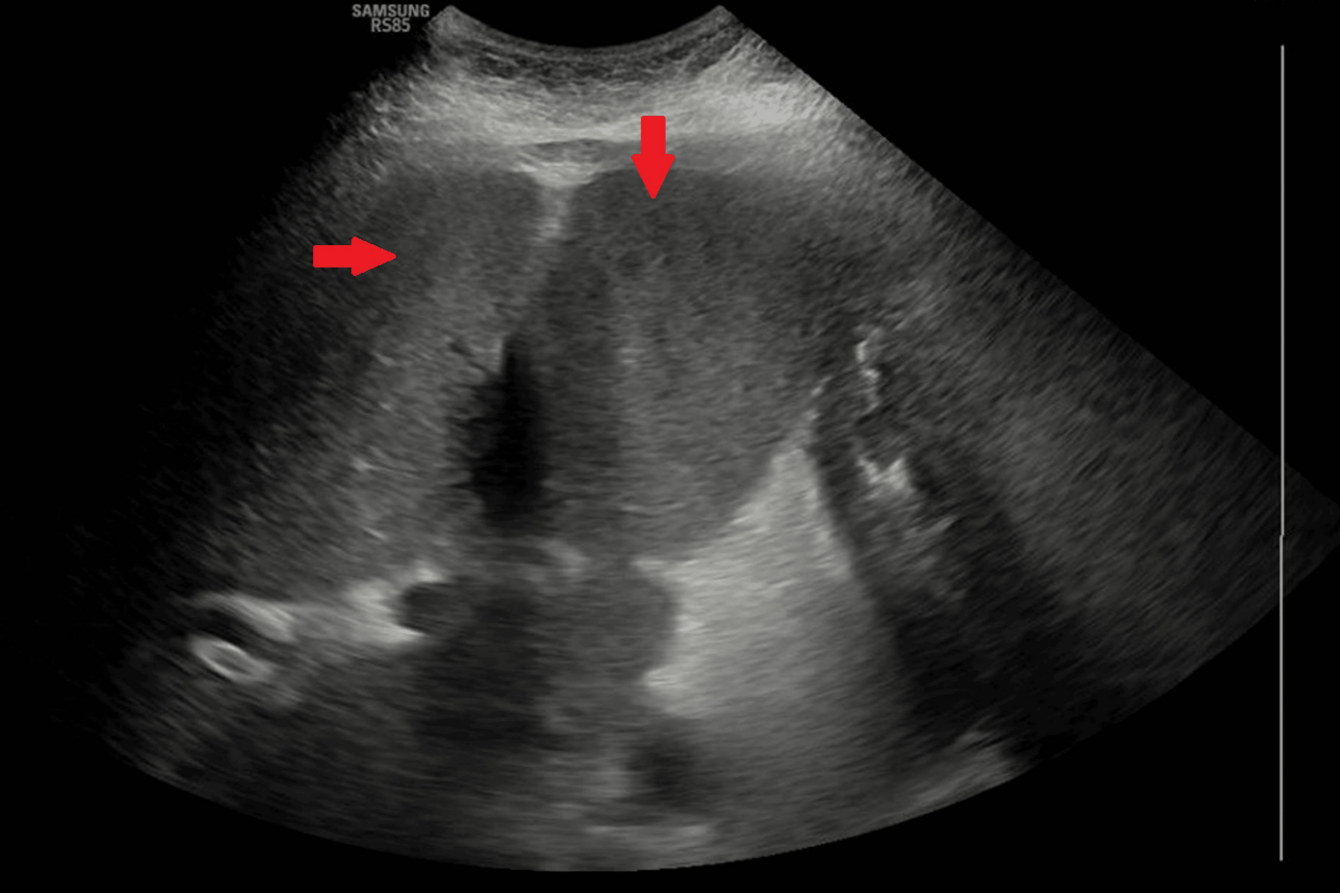
Ultrasound (US) of the liver Liver ultrasound showing homogeneous echotexture with smooth margins and with no evidence of malignancy

**Figure 4:**
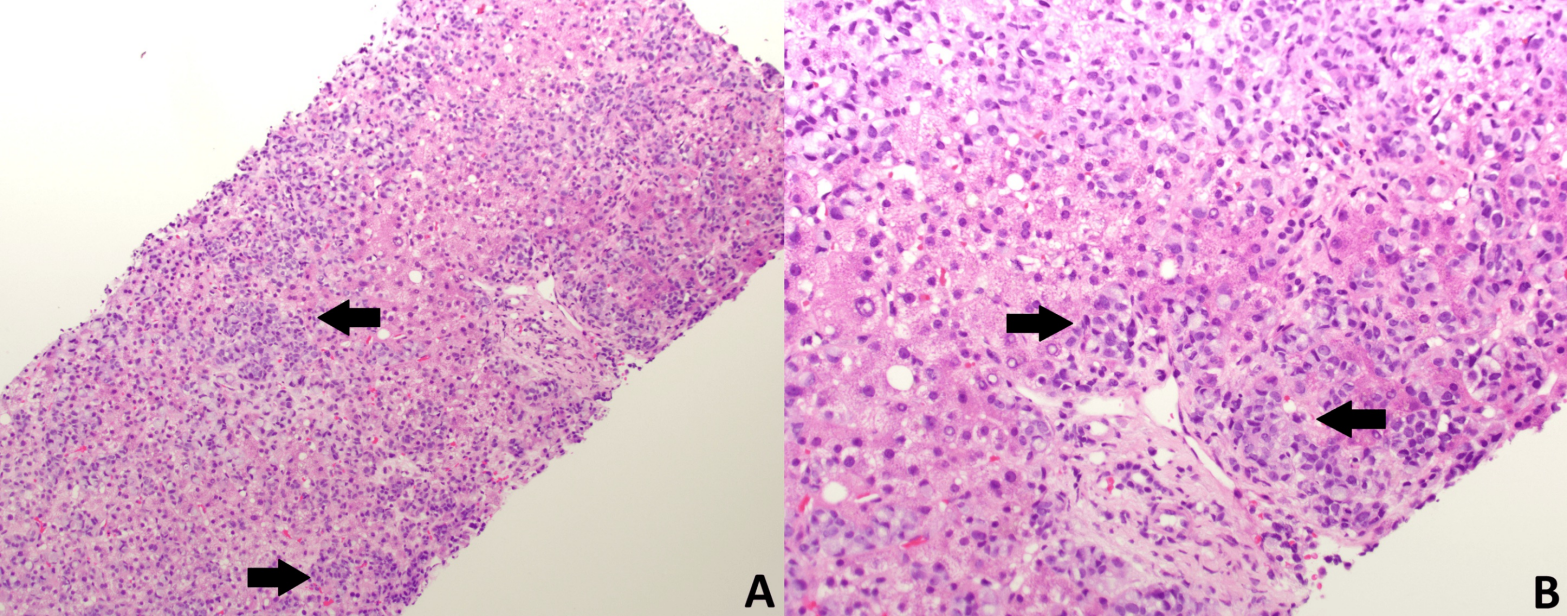
Hematoxylin and Eosin (H&E) staining of a liver core biopsy at 10x magnification (A) and 20x magnification (B) Medium-power view of a liver needle core biopsy showing a diffuse sinusoidal infiltrate of tumor cells (dark blue cells, marked with black arrow) within the liver parenchyma (pink cells). Tumor involvement extends through the sinusoidal spaces and portal tracts in a diffuse pattern, with no discrete mass or well-circumscribed nodule observed

**Figure 5:**
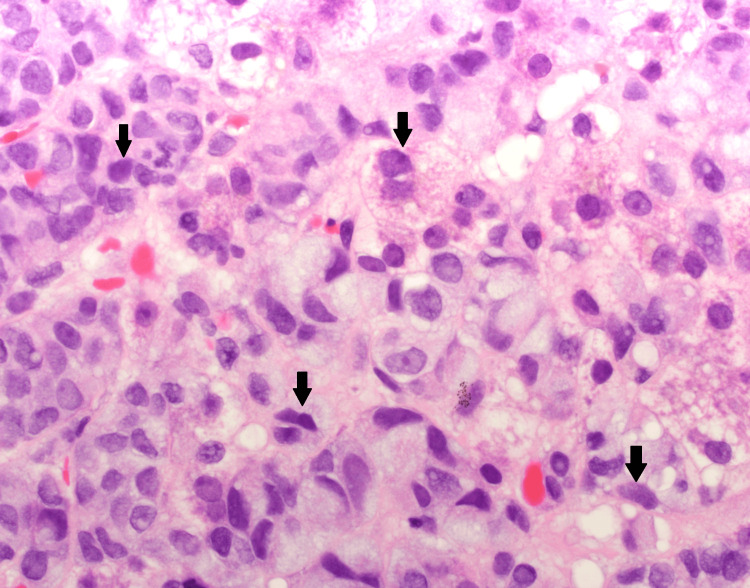
Hematoxylin and Eosin (H&E) staining of a liver core biopsy at 40x magnification High-power image depicting tumor cells with hyperchromatic, irregular nuclei and intracellular mucin globules (black arrow). Hepatocytes are identified by their pink cytoplasm, round nuclei with central nucleolus, and abundant eosinophilic cytoplasm containing green-brown bile pigment

A 56-year-old female with a history of metastatic breast adenocarcinoma, on fulvestrant (estrogen receptors blocker) and palbociclib (cyclin-dependent kinases inhibitor), presented to the emergency department with nausea, vomiting, abdominal pain, and jaundice. Her vital signs showed temperature of 37.0°C (reference range: 36.5°C-37.3°C), heart rate of 85 beats per minute (reference range: 60-100 beats per minute), respiratory rate of 16 breaths per minute (reference range: 12-18 breaths per minute), blood pressure of 135/85 mmHg (reference range: 90/60-120/80 mmHg), and oxygen saturation level of 95% on ambient air (reference range: 95%-100%). The physical examination was notable for jaundice in the skin and scleral icterus. Additionally, there was tenderness in the right upper quadrant and epigastric region, accompanied by a positive Murphy's sign. Admission labs are included in Table 1 and were significant for elevated total bilirubin, direct bilirubin, alkaline phosphatase (ALP), alanine aminotransferase (ALT), and aspartate aminotransferase (AST). The CT scan of the abdomen with contrast, performed upon admission, demonstrated evidence of cholelithiasis accompanied by gallbladder wall thickening and adjacent edema, suggesting acute cholecystitis. However, no intrahepatic or extrahepatic biliary dilatation was noticed on the CT scan (Figure 1). The liver was unremarkable, and the biliary tree exhibited a standard caliber. In light of the imaging findings consistent with acute cholecystitis and to exclude the possibility of choledocholithiasis, a magnetic resonance cholangiopancreatography (MRCP) and hepatobiliary iminodiacetic acid scan (HIDA scan) were conducted. The results of the MRCP and HIDA scan were deemed unremarkable (Figure 2). Since imaging studies ruled out the presence of choledocholithiasis and calculous cholecystitis, endoscopic retrograde cholangiopancreatography (ERCP) and cholecystectomy were not indicated. The patient's symptoms were minimally managed with conservative treatments, including antiemetics and analgesics. Her direct bilirubin levels continued to uptrend and reached 14.3 mg/dl on day five of admission. However, her ALP, ALT, and AST were stable, with minimal to no change at that time (Table 1). A comprehensive viral hepatitis panel was conducted to assess the etiology of the cholestatic liver injury, which included testing for hepatitis B surface antigen (HBsAg), hepatitis B core antigen (HBcAg), hepatitis A antibody (anti-HAV), and hepatitis C antibody (anti-HCV). Serological tests for cytomegalovirus (CMV) and Epstein-Barr virus (EBV) were also performed. In parallel, an autoimmune panel was obtained, comprising anti-nuclear antibodies (ANA), antimitochondrial antibodies (AMA), and anti-smooth muscle antibodies (ASMA). The results from these assessments were all negative. An ultrasound-guided two-core liver biopsy was performed on day six of admission due to an unexplained increase in direct bilirubin to 15.8 mg/dL (Figure 3). She was also noted to have a small amount of ascites and required diagnostic ultrasound-guided paracentesis, which was performed by the Interventional radiology team in the right lower quadrant. Ascitic fluid analysis was significant for SAAG >1.1 g/dL and ascitic protein <3 g/dL, suggestive of portal hypertension etiology. Ascitic fluid cytology revealed no malignant cells, as immunohistochemistry staining for BerEP4, MOC31, estrogen receptor, progesterone receptor, GATA3, and mammaglobin was negative. The paracentesis was complicated by hemorrhagic shock due to bleeding from the right superficial circumflex iliac artery, which triggered disseminated intravascular coagulation (DIC). She required angiogram embolization by interventional radiology (IR), intensive care unit (ICU) transfer, blood transfusion, and vasopressors. She was not a surgical candidate, as any surgical intervention would have caused massive bleeding and death. She passed away on day seven of admission. Eventually, the liver biopsy confirmed poorly differentiated metastatic lobular breast carcinoma. Both core biopsies demonstrated diffuse sinusoidal infiltrate of poorly differentiated tumor cells, with intracytoplasmic features consistent with lobular carcinoma metastasis. Iron stains revealed a focal presence of scant iron. No abnormal PAS-positive material was identified within the hepatocytes on PAS stain, with or without diastase digestion. Trichrome and reticulin stains showed fibrosis associated with tumor metastasis (Figure 4, Figure 5). It is noteworthy that a FDG PET scan conducted one month before admission revealed lytic bone lesions; however, the remainder of her body imaging appeared relatively unremarkable. The imaging indicated hepatomegaly, but no focal lesions were identified, and the gallbladder also appeared normal.

**Table 1:** Liver function panel trend during the patient's hospital course

Analyte	Day 1	Day 3	Day 5	Day 6	Reference range
Alanine aminotransferase (ALT)	48 IU/L	39 IU/L	38 IU/L	37 IU/L	7-52 IU/L
Aspartate aminotransferase (AST)	115 IU/L	100 IU/L	113 IU/L	134 IU/L	13-39 IU/L
Alkaline phosphatase (ALP)	145 IU/L	133 IU/L	179 IU/L	157 IU/L	34-104 IU/L
Total bilirubin	9.8 mg/dl	12.1 mg/dl	20.5 mg/dl	22 mg/dl	0.3-1.0 mg/dl
Direct bilirubin	6.4 mg/dl	N/A	14.3 mg/dl	15.8 mg/dl	0.0-0.2 mg/dl

## Discussion

The incidence of breast cancer metastasis to the liver is 1.4% among newly diagnosed breast cancer patients and 29.6% among those with metastatic disease [9]. Cholestatic liver injury is not a typical manifestation of breast cancer metastasis to the liver, which usually presents as a mixed hepatocellular and cholestatic liver injury pattern [10]. In our case, the R Factor for Liver Injury score was 1.0, indicating cholestatic injury [3]. The absence of any imaging findings to indicate liver involvement in our patient made the diagnosis particularly challenging. Breast cancer primarily metastasizes to the liver through the activation of liver sinusoidal endothelial cells by breast cancer-derived extracellular vesicles, which induce endothelial to mesenchymal transition and disrupt vessel barriers, facilitating cancer cell adhesion and extravasation. 

Sinusoidal tropism in breast cancer cells is facilitated by chemokine receptors such as CXCR4 and CCR7, which mediate chemotaxis toward their ligands CXCL12 and CCL21, respectively, and adhesion molecules like E-selectin and CD44, which enhance cell adhesion and migration under physiological flow conditions [11,12]. Liver metastasis typically presents as multiple distinct hypovascular or hypervascular masses; it is rare for it to present as diffuse infiltrating neoplasms that are undetected in radiologic evaluation studies [13]. These metastases often appear as hypoenhancing lesions on CT scans, hypoechoic lesions on ultrasound, and hypointense on T1-weighted MRI while being hyperintense on T2-weighted MRI with characteristic peripheral ring enhancement during the arterial phase [14,15]. 

When it comes to detecting diffuse intrasinusoidal metastatic liver disease from breast cancer, ultrasound has a sensitivity of 55% and specificity greater than 85%, CT scans have a sensitivity of 66-72% and specificity greater than 85%, MRI has a sensitivity of 76-95% and specificity greater than 85%, and PET scans show a sensitivity of 87-95% and specificity of 83-97% [16]. All aforementioned imaging modalities were conducted and revealed no signs suggestive of metastatic liver disease. However, poorly differentiated carcinoma diffusely infiltrating hepatic sinusoids may rarely present as radiographically occult in imaging studies instead of forming distinct masses, including CT, US, and MRI, due to the absence of discrete mass lesions. This pattern can lead to cryptogenic liver failure and may not display the typical imaging features [17,18].

A literature review revealed a few cases similar to our patient's where complications of liver metastasis were evident well before imaging findings were uncovered [18,19]. Allison et al. presented three cases of metastatic breast cancer to the liver that were not visible on imaging. Two of these patients had a prior diagnosis of infiltrating ductal carcinoma of the breast and presented with new-onset liver failure. The third case had no history of carcinoma but presented with severe thrombocytopenic thrombotic purpura-like syndrome, with metastatic carcinoma of the breast diagnosed only at autopsy [18]. In another instance, a patient with breast cancer quickly developed liver failure that was later confirmed to be secondary to intrasinusoidal spread, despite previous CT abdomen and Doppler ultrasound of the liver showing hepatomegaly, hepatic infarcts, and compression of the intrahepatic inferior vena cava instead of typical metastasis [19]. This diffuse intrasinusoidal metastatic pattern is most often reported in blood cancers but can also be associated with solid tumors, including small-cell lung cancer, breast cancer, and gastric cancer [20]. It is important to note that while evaluating the patient, the culprit was presumed to be DILI, considering that the patient was actively receiving fulvestrant and palbociclib three weeks before admission, both of which have reported DILI as a potential side effect [5]. Because DILI is a diagnosis of exclusion, a comprehensive workup was necessary to confirm or rule out the diagnosis. Furthermore, the rapid increase in bilirubin levels necessitated an extensive workup, culminating in a liver biopsy. This complexity added to the difficulty of the diagnosis.

## Conclusions

This case describes a rare instance of breast adenocarcinoma presenting as cholestatic liver injury and significant hyperbilirubinemia, with no radiological evidence of liver metastases on ultrasound, CT, MRI, or PET scans. The absence of these findings complicated the diagnosis, particularly with differential diagnoses such as DILI. For cancer patients with cholestatic liver injury and negative initial imaging (including CT scan and ultrasound), laboratory tests are essential to evaluate for DILI and autoimmune or viral hepatitis. If results are inconclusive, advanced imaging like MRI with liver protocol and MRCP should be conducted to detect subtle malignancies and biliary abnormalities. Additionally, an FDG PET scan should be considered in cases with high clinical suspicion. If these imaging studies are also non-diagnostic, a liver biopsy may be necessary to assess for infiltrative diseases or other liver conditions. In this case, the patient later developed DIC and hemorrhagic shock after a diagnostic paracentesis, resulting in the patient's mortality. Ultimately, a liver biopsy indicated poorly differentiated metastatic lobular breast carcinoma, likely resulting from diffuse infiltrative liver disease causing radiographically occult metastasis. This case underscores the necessity for physicians to consider liver metastases in cancer patients, regardless of the primary cancer site, who present with cholestatic liver injury, even when initial imaging shows no signs of metastases.
